# Novel Titania Nanocoatings Produced by Anodic Oxidation with the Use of Cyclically Changing Potential: Their Photocatalytic Activity and Biocompatibility

**DOI:** 10.3390/nano8090712

**Published:** 2018-09-11

**Authors:** Aleksandra Radtke, Monika Bal, Tomasz Jędrzejewski

**Affiliations:** 1 Faculty of Chemistry, Nicolaus Copernicus University in Toruń, Gagarina 7, 87-100 Toruń, Poland; monikaventabal@gmail.com; 2 Nano-implant Ltd., Gagarina 5/102, 87-100 Toruń, Poland; 3 Faculty of Biology and Environmental Protection, Nicolaus Copernicus University in Toruń, Lwowska 1, 87-100 Torun, Poland; tomaszj@umk.pl

**Keywords:** titanium dioxide, anodic oxidation, Ti6Al4V, biointegration, photocatalytic properties

## Abstract

The anodic oxidation of Ti6Al4V substrate surfaces with the use of cyclically changing potential enabled the production of novel titania nanocoatings. Morphologically different coatings were prepared in the ethylene glycol-based electrolyte with diluted HF and various amounts of water. The structure of the produced surface materials was characterized by X-ray diffraction, Raman spectroscopy, and diffuse reflectance infrared Fourier transform spectroscopy. Their morphology was visualized by scanning electron microscopy. The photocatalytic efficiency of produced materials was studied by the observation of methylene blue degradation under UV irradiation. Their biointegration properties were established on the basis of immunological assays, which checked murine fibroblasts adhesion and proliferation on novel coatings. The obtained results pointed out to both high biocompatibility, as well as the photoactivity of one of the obtained nanocoatings, allowing trusting in the applicative nature of this material.

## 1. Introduction

When nanoporous and nanotubular titania-based materials were produced as a result of electrochemical processes, many completely different TiO_2_ nanomaterials were discovered and characterized [1,2,3,4]. Their number is constantly increasing due to very easy modification of the anodization processes by changing the type and composition of the used electrolyte solution, the applied current potential, the temperature, and the anodization time. Materials based on titanium dioxide and produced in electrochemical processes are the topic of intense studies and their unique physicochemical and biological properties are used in numerous branches of industry. For example, good biointegration activity of TiO_2_ materials in combination with the resistance towards body fluid effects and high corrosion resistance means that titania is one of the best materials for the modification of titanium implant surfaces [5,6]. Furthermore, tubular TiO_2_ can reveal an excellent photocatalytic activity in the range of UV radiation, so they can be used in implant sterilization before its application [5,7].

The electrochemical anodization process is one of the easiest ways to prepare TiO_2_ nanomaterials, especially titania nanotubes. The electrochemical cell used in the synthesis contains a titanium/titanium alloy anode and a platinum cathode immersed in the diluted fluoride electrolyte and connected to the DC power source for a given time [3]. The primarily TiO_2_ grows as a result of the titanium/titanium alloy sheet’s electrochemical oxidation. Further, because of the presence of fluoride ions, the compact titania is etched and nanostructures are formed [6]. Actually, the research on TiO_2_ nanotubes is classified into four generations. Zwilling et al. [2] have reported the formation of nanoporous anodized titania and Gong et al. [8] have commented work on anodized titania nanotubes by using a dilute solution of hydrofluoric (HF) acid as an electrolyte. This kind of titania nanotubes is known as first generation nanotubes. The thickness of obtained anodic layer was limited up to 0.5 µm because of using HF, which etched and dissolved most of the growing oxide. When HF acid was replaced with less aggressive solutions containing fluoride salts, the rapid rate of titania dissolution was reduced and the thickness of the reported anodic layer was 2–3 µm [9,10,11,12]. This kind of titania nanotubes is known as second generation nanotubes. Even if the thickness of second-generation nanotubes has been improved, the use of water yielded irregularities along the walls of the nanotubes. Because of this fact, studies on smooth tubes without ripples along the wall were done and it has been found that such kind of titania nanotubes, known as third generation nanotubes, can be prepared in organic electrolytes. Macak et al. have reported the formation of titania nanotubes by using organic electrolytes and found an anodic layer thickness about 7 µm [13]. Actually, third-generation electrolytes, like a gliceryne or an ethylene glycol containing a diluted hydrofluoric acid or its ammonium or potassium salts are the most popular [14,15,16,17]. With their participation, obtained structures can be longer and better ordered, and their morphology control is simply based on the changing of the anodization parameters. In case of third-generation electrolytes, one of the easiest ways to control and change titania coating morphology is changing the water percentage in them. Water content variations of around 1–2%, give radical changes in the length and appearance of the surface topography. Synthesis of TiO_2_ nanotube arrays using fluoride-free electrolytes is commonly considered a fourth generation synthesis technique. The non-fluoride-based electrolytes include HCl, H_2_O_2_, and their mixtures, perchloric acid solutions, and mixtures of oxalic acid, formic acid, and sulfuric acid with NH_4_Cl [18].

As it was mentioned, the electrochemical anodization synthesis is considered to be a popular one due to its good controllability on structural properties. Feng Zhou et al. [19] have reported the synthesis of titania nanotubes with tunable morphologies by adjusting the reaction conditions during anodization. Changing the reaction temperature, applied voltage and HF concentration obtained either nanoporous TiO_2_ nanotubes or free-standing nanotubes with a tunable pore size, length and wall thickness. Schmuki et al. [20,21] have reported the growth of TiO_2_ nanobamboo tubes by using alternating-voltage anodization of Ti in fluoride-containing electrolytes. They have found that using a simple variation of the electrochemical conditions, the geometry and surface properties of the nanotube layers can be altered over a wide range.

The wide use of titanium alloys (especially Ti6Al4V) in the production of implants, and the necessity to solve problems related to the optimal connection of a metal implant with the bone (osteointegration) has caused interest in the use of anodization processes to modify their surface [6,7,22]. Previous studies have revealed that the anodization of Ti6Al4V substrate surfaces using 0.3% HF solution as an electrolyte led to obtaining coatings of TiO_2_ nanotubes (TNT), the diameters of which depended on the used potential (e.g., 3–20 V) [7,22,23]. Titania nanotube layers were also produced at higher potentials (30–100 V), using glycerine or ethylene glycol-based electrolytes with fluoric acid or its ammonium/potassium salts [14,15,16,17]. Looking for new coatings of better biointegration properties compared to the systems currently used, we paid attention to the possibility of modifying the anodization process using a cyclically changing potential during the electrochemical oxidation process of the Ti6Al4V alloy surface. This led to obtaining titanium dioxide coatings with atypical architecture, which prompted us to assess their biointegration properties as well as photocatalytic activity. Analysis of previous literature reports showed that this type of research mainly relates to TNT coatings of different size, structure, and properties. There are relatively few reports about titania coatings with different nanotubular morphology, produced during the anodic oxidation process. Therefore, in this article we decided to present the results of our work (especially the assessment of the properties of biological activities) of TiO_2_ coatings with an unusual architecture.

## 2. Materials and Methods

### 2.1. Synthesis of TiO_2_ Nanomaterials: Porous Titania Nanocoatings; Characterization of Their Structure and Their Morphology

The studied titania coating materials were produced during the electrochemical anodization process on the surface of 5 × 70 mm titanium alloy (Ti6Al4V) samples, which acted as anode. A platinum wire (99.99%) was used as a cathode. The distance between the electrodes was equal to 2 cm. Before the oxidation process, Ti6Al4V samples were cleaned by ultrasonication for 15 min in acetone, ethanol, distilled water, and dried in an Argon stream. Then, samples were chemically etched in a 1:4:5 mixture of HF:HNO_3_:H_2_O for 30 s and after rinsing in deionized water and drying they were introduced into the electrolyte solution. The main electrochemical processes were carried out at room temperature, using ethylene glycol as a base electrolyte with 0.2 M hydrofluoric acid and variable amounts of water (0.3, 1, and 2 wt %). The anodic oxidation of the surface of Ti6Al4V samples has been performed using a two-stage procedure with cyclically changing potential. The first stage included six cycles of changes between 30 V and 10 V, both for 5 min. In the second stage, six cycles of 30 V for 50 s and 0 V for 10 min were used. Finally, the titanium alloy substrate was washed with distilled water and dried in an Argon stream. In order to make comparisons of the properties of novel coatings with already known titania nanotube coatings, the standard anodic oxidation of Ti6Al4V in ethylene glycol as a basis electrolyte with 0.2 M hydrofluoric acid and 1 wt % of water, under the constant potential −30V, has been carried out. The morphology changes of the sample surfaces were studied using Quanta field-emission gun Scanning Electron Microscope (SEM; Quanta 3D FEG; Carl Zeiss, Göttingen, Germany). The structure of the produced TiO_2_ nanomaterials was analysed using X-ray diffraction (PANalytical X’Pert Pro MPD X-ray diffractometer, PANalytical B.V., Almelo, The Netherlands), using Cu-Kα radiation and grazing incidence angle mode-GIXRD; the incidence angle was equal to 1 deg), diffuse reflectance infrared Fourier transform spectroscopy (DRIFT, Spectrum 2000 PerkinElmer, Perkin Elmer Corporation, Norwalk, CT, USA), and Raman spectroscopy (RamanMicro 200 PerkinElmer, Waltham, MA, USA).

### 2.2. Photocatalytic Activity Studies of the Produced TiO_2_ Nanocoatings

The photocatalytic activity of the produced titania coatings was estimated using the most frequently descriptive procedure in the literature, i.e., methylene blue (MB, 3 cm^3^) photodegradation [7]. In all our experiments, we were waiting 30 min for the adsorption equilibria after the samples of the porous materials were immersed in the aqueous 10^−5^ M methylene blue solution (in the darkness), and then they were illuminated by UV radiation (18 W, range of 340–410 nm with maximum at 365 nm). The changes in MB concentration were registered every 24 h for seven days by spectrophotometric analysis (Metertech SP-830 PLUS, Nangang, Taipei, Taiwan). The starting point for kinetic calculations is the method of chemical kinetics assumption a Langmuir–Hinshelwood reaction mechanism. If we assume a low initial concentration of MB, the photodegradation process can be treated as a reaction with pseudo-first-order kinetic, which is described by the equation:

*c_t_* = *c*_0_ exp(−*k*_obs_*t*) (1)

where, *c*_0_—initial concentration of MB, *c_t_*—MB concentration after time *t*, *k*_obs_—the observable rate constant.

During calculations, the blind tests (degradation without UV and titania samples) were taken into account.

### 2.3. Biocompatibility Studies of Titania Nanocoatings: The Adhesion and the Proliferation of L929 Cells on the TiO_2_ Nanocoatings

Murine fibroblast cells (L929; American Type Culture Collection) were cultured in the same culture conditions as we have presented in earlier reports [22,23]. The effect of the produced porous titania coatings as well as the titania nanotubes TNT30 on the adhesion (after 24 h) and proliferation (after 72 h and 120 h) of L929 cells were assessed using the MTT (3-(4,5-dimethylthiazole-2-yl)-2,5-diphenyl tetrazolium bromide; Sigma Aldrich, Darmstadt, Germany) assay. Statistical significance in the MTT assay was determined using one-factor analysis of variance (ANOVA). As a post hoc test, the Tukey test was used. The level of significance was set at *p* < 0.05. The changes in the morphology of murine fibroblasts cells growing on the surface of titania nanocoatings were observed using Scanning Electron Microscopy (SEM, Quanta 3D FEG; Carl Zeiss, Göttingen, Germany) using the same method as in [22,23].

## 3. Results

**Figure 1:**
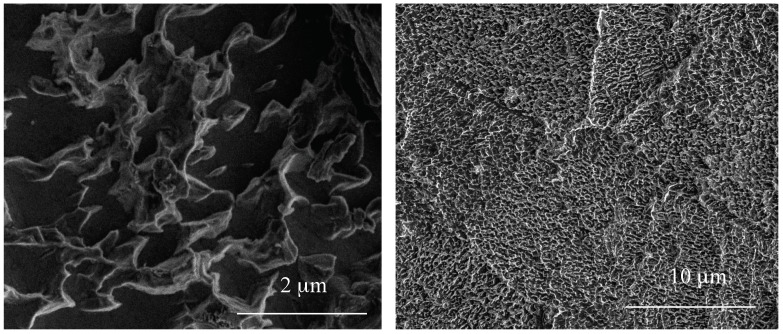
Scanning Electron Microscope (SEM) images of TiO_2_ porous material (PTN) produced in the variable potential two step anodization process in ethylene glycol-based electrolyte obtained on Ti6Al4V alloy surface.

The applied electrochemical oxidation technique led to the formation of different types of porous coatings on the Ti6Al4V substrate surface. Analysis of SEM images revealed that anodization of sample surface, using the ethylene glycol as an electrolyte with 0.2 M hydrofluoric acid and 1 wt % of water, caused that substrate surface to be uniformly covered with coating composed of vertically arranged bungs with not very well-defined shapes resembling ribbons between which there were pores with diameters in the range of about 300–800 nm (Figure 1). For the purposes of this article, this type of material was called porous titania coating (PTN).

**Figure 2:**
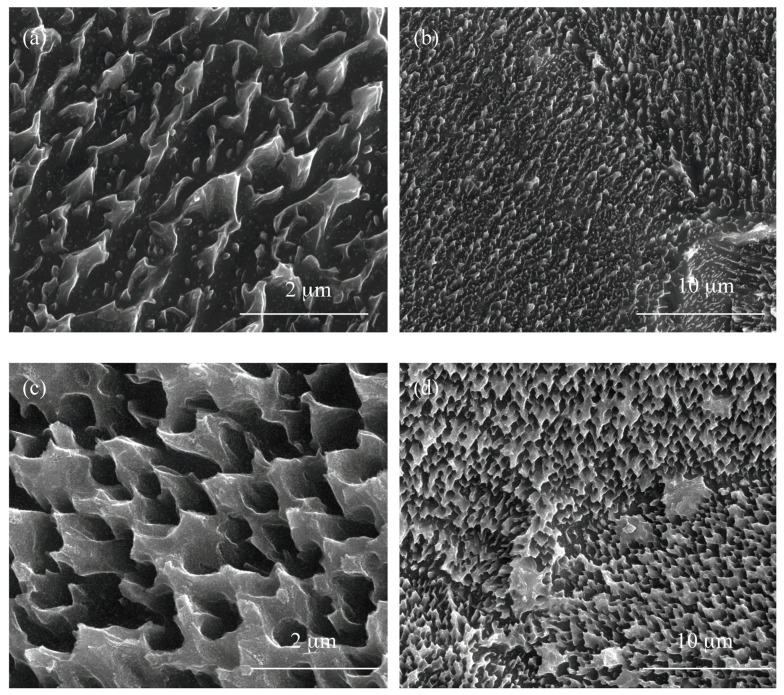
Titania nanoteeth coatings produced in the variable-potential two-step anodization process in ethylene glycol-based electrolyte with different amount of water: 0.3 wt % (**a**,**b**) (TNTE03); and 2 wt % (**c**,**d**) (TNTE2).

SEM images of materials produced using the electrolyte solution containing 0.3 and 2 wt % of water are presented in Figure 2. In this case, the surface layer morphology significantly depends on the water content in the electrolyte solution, i.e., 0.3 and 2 wt %. The slight water content (0.3 wt %) in the electrolyte led to the formation of a coating composed of scattered TiO_2_ grains in the shape of teeth, therefore this type of the material we have called as a titania nanoteeth coating (TNTE03, Figure 2a,b). By increasing the water content in the electrolyte solution up to 2 wt % and under the same current-voltage conditions, porous layers composed of teeth-like structures forming an architecture similar to TNTE03, but much more dense (Figure 2c,d). We have called this material a titania nanoteeth coating TNTE2.

**Figure 3:**
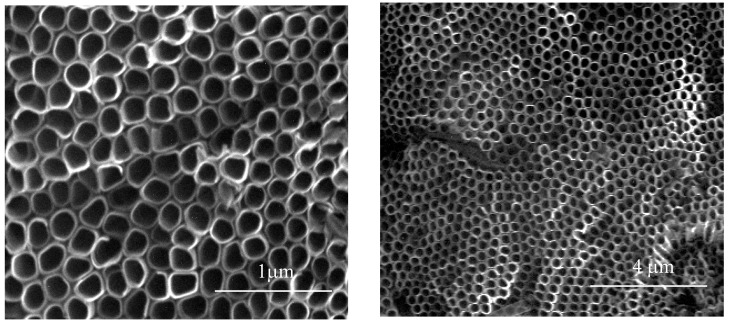
Titania nanotubes coating (TNT30) produced in ethylene glycol as a basis electrolyte, which contain 0.2 M hydrofluoric acid and 1 wt % of water, under the constant potential −30 V.

SEM images of the reference titania nanotubes coatings obtained in the standard anodic oxidation procedure of Ti6Al4V in ethylene glycol as a basis electrolyte with 0.2 M hydrofluoric acid and 1 wt % of water, under the constant potential −30 V, during 1 h are presented in Figure 3.

**Figure 4:**
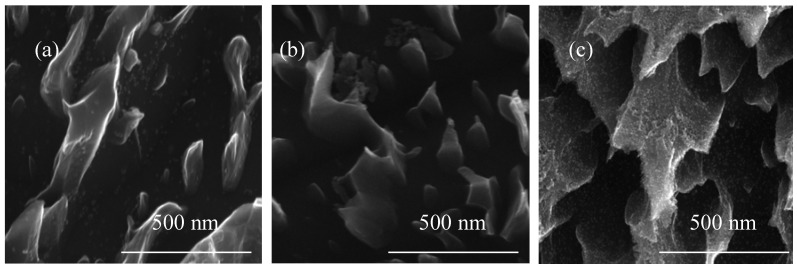
SEM images of PTN (**a**); TNTE03 (**b**); and TNTE2 (**c**) made in the form of side view.

Looking at SEM images of PTN, TNTE03, and TNTE2, especially at lower magnifications, one can get the impression that we are not dealing with a layer on the surface, but with pits. However, a detailed analysis of SEM images shows that on the surface of the titanium alloy there is a chemically distinct system morphologically reminiscent of ribbons, teeth and protrusions (Figure 4).

### 3.1. Structural Characterization of Produced Nanomaterials

**Figure 5:**
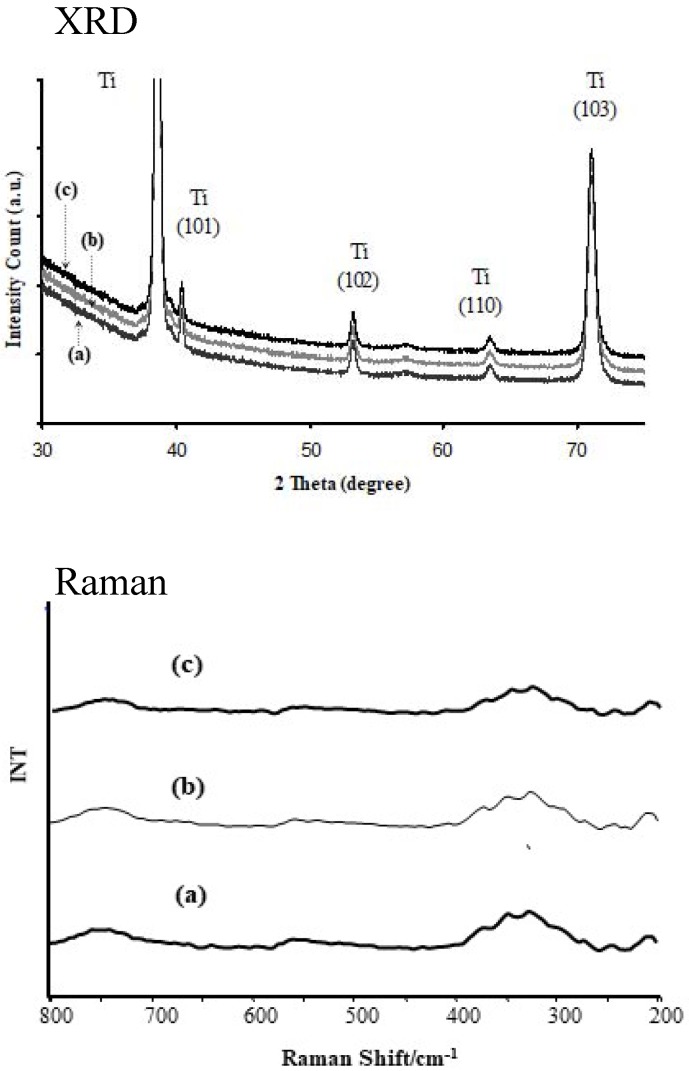
XRD and Raman spectra of produced titania materials: (**a**) PTN; (**b**) TNTE03; (**c**) TNTE2.

The structure of morphologically different materials (PTN, TNTE03 and TNTE2) was studied using X-ray diffraction and Raman spectroscopy. The results of these investigations are presented in Figure 5. We expected to obtain a titanium dioxide layer in the process of titanium alloy anodic oxidation. Analysis of the obtained data proved that materials produced on the surface of Ti6Al4V substrates, using the two-step periodically variable potential anodization process, were amorphous. Fingerprints of crystalline titanium dioxide could be seen neither in the XRD patterns nor in the Raman spectra [24,25].

**Figure 6:**
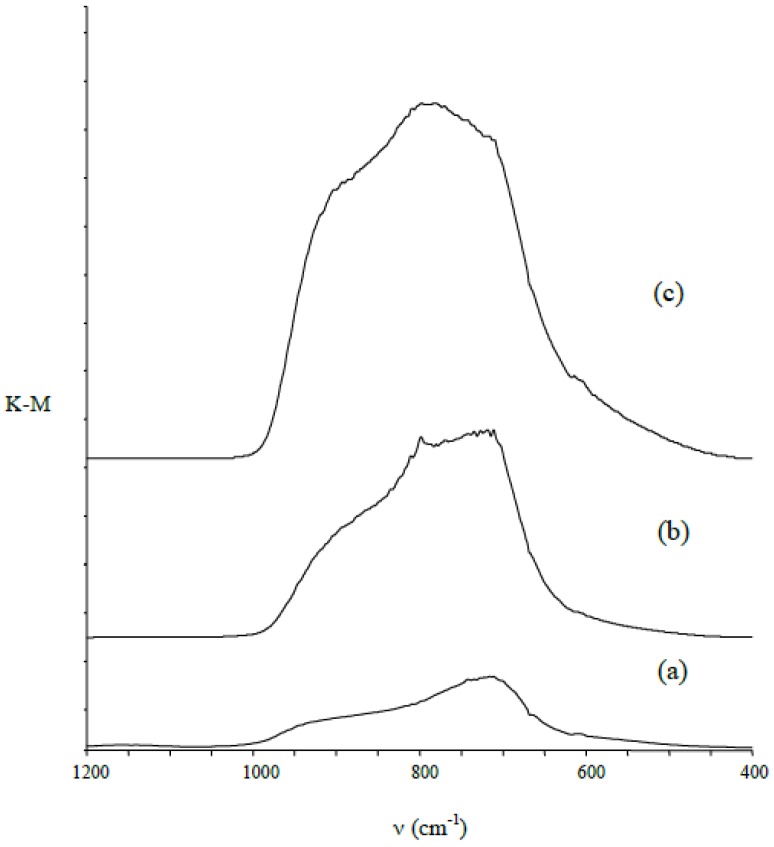
Diffuse reflectance infrared Fourier transform spectroscopy (DRIFT) spectra of the produced titania materials: (**a**) PTN; (**b**) TNTE03; (**c**) TNTE2.

However, in order to confirm the presence of TiO_2_ on the surface of the titanium alloy, we have used diffuse reflectance infrared a Fourier transform spectroscopy (DRIFT) method. In these spectra, in all cases we found an intensive absorption band in the range of 750–890 cm^−1^, which can be attributed to lattice TiO_2_ modes: ν(Ti–O) and δ(Ti–O) [7,26,27,28]. This band confirms the formation of a TiO_2_ layer on the substrates surface (Figure 6).

### 3.2. Studies on the Photocatalytic Properties of Obtained Nanomaterials

Photocatalytic activity of the produced materials was evaluated on the basis of methylene blue (MB) photodegradation studies, which are well known and thoroughly described in the literature [7,29,30,31]. Speed of the decomposition of MB in the presence of probably the photocatalytic material compared with its photodegradation without the additional factors gives information about strength of the photocatalytic properties of the studied substance. Based on Equation (1), given in Materials and Methods, the observable rate constant of the MB degradation process has been calculated, and the obtained results are listed in the Table 1. The titania nanotube coating (TNT30) produced on the surface of the Ti6Al4V substrate using a constant potential of 30 V has been used as a reference sample. Analysis of data presented in Table 1 revealed a significant increase of *k*_obs_ values calculated for the produced porous coatings in comparison to the reference sample (TNT30) and the blind test. The studies have shown that the registered differences in photocatalytic activity of the produced materials are associated with differences in their surface morphology.

### 3.3. The Evaluation of Biointegration Properties of the Produced Titania Layers

**Figure 7:**
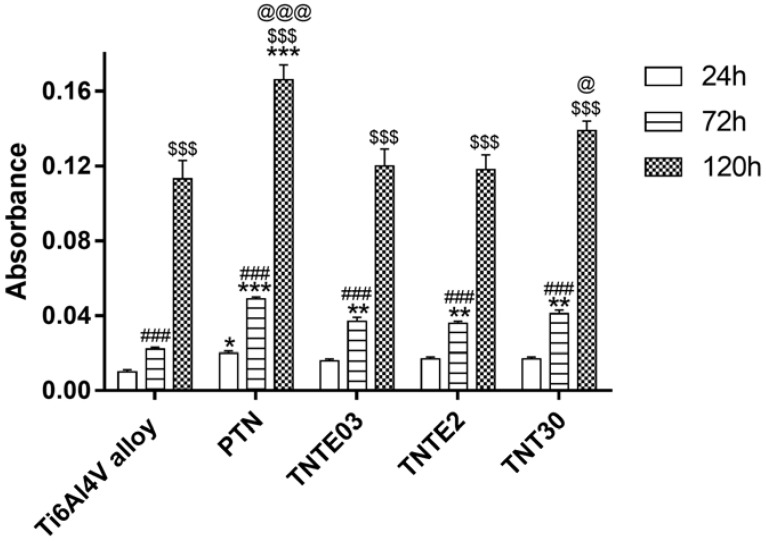
Effect of the tested nanocoatings on the murine fibroblasts L929 adhesion (after 24 h) and proliferation (after 72 h and 120 h) detected by MTT assay. The absorbance values are expressed as means ± S.E.M. of three independent experiments. Asterisks indicate significant differences between the fibroblasts incubated with Ti6Al4V alloy references sample compared to the porous titania nanocoating (PTN), titania nanoteeth (TNTE03 and TNTE2) and the reference sample of titania nanotube coating (TNT30) at appropriated incubation time (* *p* < 0.05, ** *p* < 0.01, *** *p* < 0.001). Hash marks denote significant differences between the cells incubated with the same tested nanomaterial for 24 h in comparison to 72 h (^###^ *p* < 0.001). $ marks indicate significant differences between the fibroblasts incubated with the specified nanomaterial for 72 h compared to 120 h incubation time (^$$$^ *p* < 0.001). @ marks denote significant differences between titania nanoteeth and TNT30 or PTN samples (^@^ *p* < 0.05, ^@@@^ *p* < 0.001).

The biocompatibility of the studied novel nanocoatings (PTN, TNTE03 and TNTE2) was evaluated based on the MTT assay results, which were related to the adhesion (measured after 24 h) and proliferation (assessed after 72 h and 120 h) of L929 murine fibroblasts. It is worth noticing that with an increase of incubation time, more L929 cells proliferated on the tested nanomaterials (Figure 7). Moreover, analysis of these data revealed that both in the case TNTE03 and TNTE2 samples, a higher level of proliferation was observed only after 72 h (*p* < 0.01) compared to the Ti6Al4V alloy. In contrast, the samples of PTN showed significantly higher levels of adhesion (*p* < 0.05) and proliferation after 72 h (*p* < 0.001) as well as 120 h (*p* < 0.001) in comparison to the reference sample (Ti6Al4V alloy). The level of adhesion (after 24 h) and proliferation (measured after 72 h) of the cells growing on the surface of the reference sample of titania nanotube coating (TNT30) was comparable to the values observed for TNTE03 and TNTE2 samples. On the other hand, after 120 h of incubation, we have noticed a slight increase in fibroblast proliferation (*p* < 0.05), which was however lower than in the case of the porous titania nanocoating.

**Figure 8:**
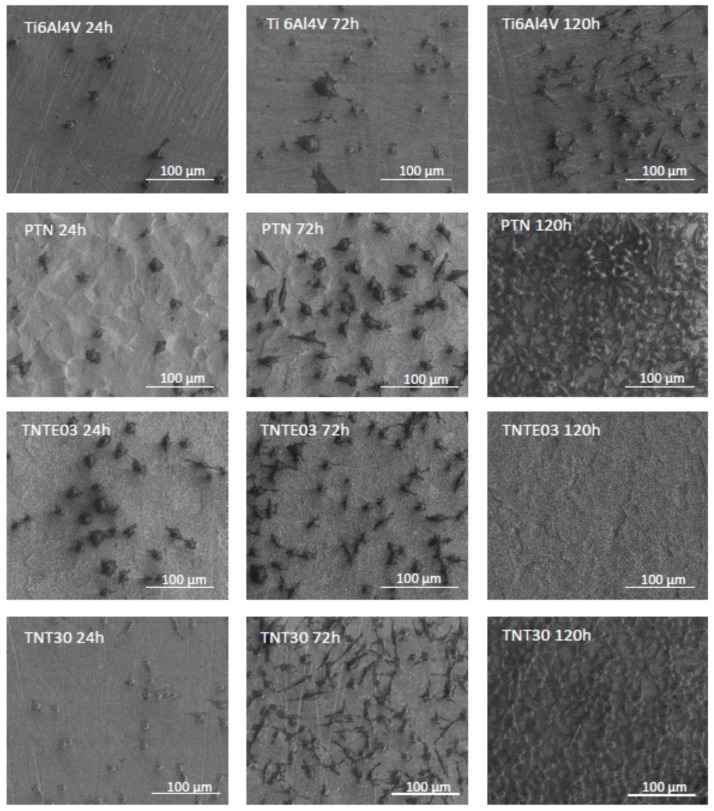

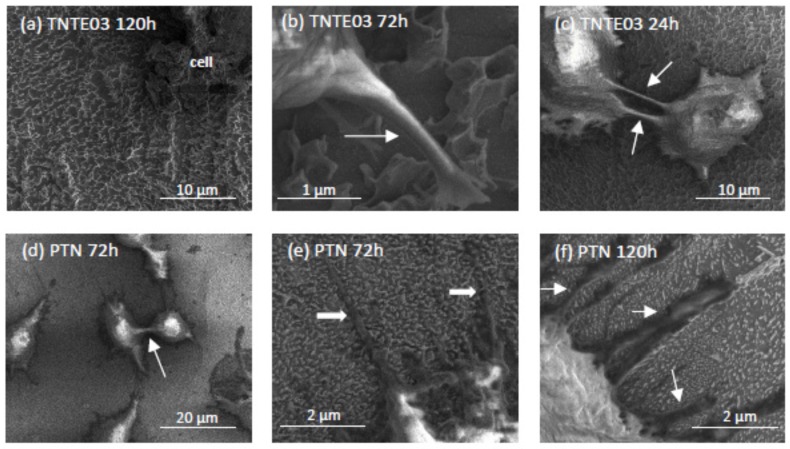
Scanning electron microscopy (SEM) images showing the murine L929 fibroblasts adhesion (after 24 h) and proliferation (after 72 h or 120 h) on the surfaces of the tested nanocoatings: Ti6Al4V alloy references sample, porous titania nanocoating (PTN), titania nanoteeth (TNTE03), and titania nanotube coating (TNT30). The white arrows in the figure indicate the filopodia spread between fibroblasts (**c**,**d**) and filopodia, which attached the cells to the nanomaterial surface (**b**,**e**,**f**). In Figure (**a**) there are the cells that morphologically resemble the structure of the TNTE03, which makes it difficult to recognize them on the surface under greater magnification (see TNTE03 120 h; scale 100 µm).

Figure 8 shows the comparative SEM images of L929 murine fibroblasts cultured on the titania nanoteeth (TNTE03), porous titania nanocoatings (PTN) and titania nanotube coating (TNT30) for 24, 72 and 120 h in comparison to the Ti6Al4V alloy references sample. These data, supporting the MTT results, clearly indicate the high level of biocompatibility of the tested biomaterials, which is confirmed by the increase in the number of cells attached to the surface of the nanomaterials over time and the formation of filopodia by the L929 cells. In the case of PTN, the fibroblasts incubated for 120 h were crowded and formed networks due to the overgrowth of cells, which indicates that the tested plates could contribute to the proliferation of the cells. This phenomenon was not observed for the Ti6Al4V alloy.

As it can be seen in Figure 8a, the cells growing on the surface of TNTE03 morphologically resemble the structure of the nanomaterial, which makes it difficult to recognize them on the surface under greater magnification (see TNTE03 120 h, scale 100 µm). Importantly, the cells growing on the tested biomaterials also formed filopodia, which attached the fibroblasts to the surface of arrays by penetrating deep into the nanolayers (Figure 8b,e,f) or formed them among themselves (Figure 8c,d).

## 4. Discussion

Electrochemical oxidation of Ti6Al4V substrate surfaces using a cyclically changing potential, in an electrolyte consisted of ethylene glycol with 0.2 M hydrofluoric acid and 0.3, 1, and 2 wt % of water, led to the formation of three morphologically different titania coatings: TNTE03, PTN, and TNTE2, respectively. The structural studies proved that all produced materials were amorphous titanium dioxide. It should be noted that using a similar type of electrolyte but maintaining a constant potential value (30 V), titania nanotube coatings (TNT30) were produced. The value of the applied potential mainly affected the tube diameters and tube wall thickness and length [32,33]. Considering previous reports of anodic oxidation it should be noted that also the water content in the electrolyte solution is the main factor affecting the morphology of the formed materials. For the electrolyte containing fluorine ions this process directly depends on the deprotonation of water molecules up to OH^−^ and O^2−^ anions. The direct reaction of these anions with Ti^4+^ leads to the formation of a TiO_2_ barrier layer on the substrate surface. The partially dissolution of this layer, controlled by the concentrations of H^+^ and F^−^ ions in the electrolyte solution and the value of used potential, results in the formation of TNT coating [34,35,36]. In the case of oxidation processes in which the glycol-based electrolytes were used, it was found that with an increase in water content in the electrolyte also its conductivity increases. This fact is associated with an increase of H^+^ and F^−^ ion diffusion rate, and also with a decrease of electrolyte viscosity. Both of the above-mentioned factors contribute to increasing the rate of chemical dissolution of the upper surface of the nanotubes and thus to the reduction of their length [37,38]. In our anodic oxidation experiments, in which the water content in the electrolyte was controlled and the potential was cyclically changed, we disturbed the balance between the formation of titanium dioxide at the oxide/substrate boundary surface and the chemical dissolution of TiO_2_ in the outer layer. For a very low content of water in the electrolyte (0.3 wt %) and cyclically changing the potential during the anodic oxidation process, self-organized anodic growth conditions were not well established, and as a result, the titania coatings composed of dispersed teeth-like grains were formed (TNTE03, Figure 2a,b). On the other hand, the use of an electrolyte solution containing a higher percentage of H_2_O-2 wt % of water caused a transition from teeth-like grains to a denser morphology, also resembling teeth, but of bigger sizes. These teeth look like unfinished “tubes”, as we can see, more regular holes 300–500 nm between the teeth (Figure 3c) and the cyclically changing potential did not allow for the properly established formation of nanotube architecture (TNTE2, Figure 2c,d). Moreover, the non-uniform outer surface layer can be explained by the chemical dissolution of the oxide layer (sharp teeth on the Figure 3c). Optimal conditions for the discussed anodic oxidation method were achieved using the addition of 1 wt % of water to the electrolyte solution (PTN). In the above conditions, the balance between the formation of TiO_2_ at the oxide/substrate boundary surface and its dissolution in the outer layer appears to be achieved. As a result, interconnected ribbons have uniformly covered the whole substrate surface between which there are pores with diameters of 500 nm (Figure 1 and Figure 3a).

Results of photocatalytic activity tests of the PTN, TNTE03, and TNTE2 coatings revealed their significantly better activity in comparison to TNT30 sample (Table 1). According to literature reports, the sizes and shapes of TiO_2_ nanostructures are important factors affecting the photocatalytic activity of titania [39,40]. Studies of TNT coatings showed the important influence of the tube length, diameter, and wall thickness. Zhuang et al. observed an increase in the efficiency of the degradation of methyl orange under the influence of UV radiation along with an increase in the length of nanotubes, reaching the maximum in the case of nanotubes with a length of 2.5 μm. Further extension of the nanotubes resulted in a decrease in their photoactivity. Simultaneously, they stated that with the increase of the nanotube diameter, the specific surface of their entire matrix is decreasing, which can affect the reduction of photocatalytic activity [41]. The photocatalytic activity increase of nanotubes with thinner walls is associated with the acceleration of the migration process of photo-excited electrons from the interior to the surface of TiO_2_, and thus with the limitation of the recombination process of charges [42]. Analysis of SEM images of TNTE2 coatings showed that coatings composed of densely packed teeth, resembling deformed tubes of diameter 300–500 nm, with wall thickness 150–300 nm, and length ca. 1 µm, form their surface. Such architecture caused photocatalytic activity of this material to be weaker than the TNTE03 system (dispersed teeth), the morphology of which allows a better migration process of photo-excited electrons from the interior to the surface of TiO_2_ and optimal access of light to the deeper parts of coatings. It is interesting that the best photocatalytic efficiency revealed PTN coatings. In this case, the PTN coating must be considered as a porous one, composed of vertically arranged bungs with not very well-defined shapes, resembling ribbons, between which there are pores with diameters in the range of 300–800 nm, which facilitate access of light to the deeper parts of the cavities, causing the acceleration of MB photodegradation process.

**Figure 9:**
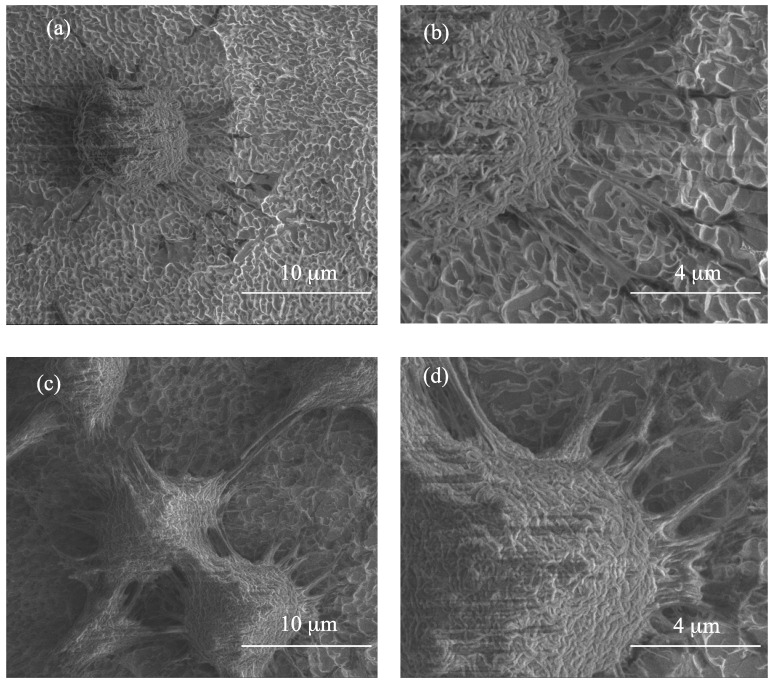
SEM images of L929 murine fibroblasts incubated on the porous titania material surface studied after 24 h (**a**,**b**) and 120 h (**c**,**d**).

The results of the MTT assays, which were related to the adhesion (measured after 24 h) and proliferation (assessed after 72 h and 120 h) of L929 murine fibroblasts revealed that PTN coatings showed not only good biointegration properties, but also absolutely unique similarity in the morphology with studied fibroblasts, which was shown in detail in Figure 9. Filopodia formation of fibroblasts is also an evidence of nanostructure biocompatibility since it has been reported that filopodia can sense the topography of biomaterial surfaces [43]. Cell filopodia play an important role in cell attachment and initiation of cell migration by sensing and transducing signals (chemical as well as mechanical) from the outside into the cell [44]. Moreover, fibroblast cells are the most common cells in connective tissue, one of the main components of peri-implant soft tissue, which is key to the formation of the peri-implant mucosal seal and helps to prevent epithelial ingrowths [45]. All of these findings taken together prove that the novel biomaterial presented in this paper (PNT) is characterized by high biocompatibility.

## 5. Conclusions

Using an electrochemical oxidation technique with cyclically changing potential and regulating the amount of water in the electrolyte, it was possible to obtain three morphologically different porous TiO_2_ coatings resembling ribbons (PTN), dispersed teeth (TNTE03), and densely packed teeth looking like deformed tubes (TNTE2). We have decided to determine their possible biointegration properties, as well as their photoactivity. The studies proved that one of the novel biomaterials presented in this paper (PTN) is characterized by high biocompatibility, caused by a unique similarity in morphology with fibroblasts. An additional advantage of this coating is its photoactivity; because of this fact, such kind of TiO_2_ architecture could be used as a biomaterial to modify implant surfaces, which can be self-sterilized in UV light.

## Figures and Tables

**Table 1:** The *k*_obs_ rate constants for MB photodegradation of blind test (without UV light and samples), and tests with porous coatings produced in glycol-based electrolyte containing HF and 0.3 wt % (TNTE03), 1 wt % (PTN), and 2 wt % (TNTE2) water. Obtained results are compared to *k*_obs_ rate constant for methylene blue photodegradation of the reference sample of titania nanotubes coating (TNT30) produced using constant potential 30 V.

Sample Rate	Constant				
	Blind Test	PTN	TNTE03	TNTE2	TNT30
10^6^ *k*_obs_ [s^−1^]	0.30 ± 0.08	2.36 ± 0.07	2.13 ± 0.06	1.98 ± 0.04	1.14 ± 0.15
